# Quenching of Tryptophan Fluorescence in the Presence of 2,4-DNP, 2,6-DNP, 2,4-DNA and DNOC and Their Mechanism of Toxicity

**DOI:** 10.3390/molecules18022266

**Published:** 2013-02-18

**Authors:** Cristina-Amalia Dumitraş Huţanu, Marius Zaharia and Olga Pintilie

**Affiliations:** Faculty of Chemistry, “Al. I. Cuza” University, 11 Carol I, RO-700506 Iasi, Romania; Tel.: +40-232201313

**Keywords:** dinitrophenols, 2,4-dinitroanisole, oxidative phosphorylation, tryptophan, fluorescence, complex, computational, electronic chemical potential

## Abstract

Although they are widely used as insecticides, acaricides and fungicides in the agriculture or as raw materials in the dye industry, dinitrophenols (DNPs) are extremely noxious, death cases having been registered. These compounds produce cataracts, lower leucocyte levels, disturb the general metabolism and can cause cancer. It is also assumed that DNPs hinder the proton translocation through the mitochondrial inner membrane and therefore inhibit oxidative phosphorylation. Their fluorescence quenching properties can help understand and explain their toxicity. Fluorescence quenching of tryptophan was tested using different dinitrophenols such as 2,4-dinitrophenol (2,4-DNP), 4,6-dinitro-orthocresol (DNOC), 2-[(2,4-dinitrophenyl)amino]acetic acid (GlyDNP), 2-(1-methyl-heptyl)-4.6-dinitrophenyl crotonate (Karathan), 2-amino-5-[(1-((carboxymethyl)amino)-3-((2,4-dinitrophenyl)thio)-1-oxopropan-2-yl)amino]-5-oxopentanoic acid (SDN GSH), 2,4-dinitroanisole (2,4-DNA) and 2,4-dinitrobenzoic acid (2,4-DNB). 2,4-DNP and DNOC showed the highest tryptophan fluorescence quenching constant values, these being also the most toxic compounds. The electronic chemical potential value of the most stable complex of 2,4-DNP-with tryptophan is higher than the values of the electronic chemical potentials of complexes corresponding to the derivatives.

## 1. Introduction

Dinitrophenols and their derivatives are used in medicine, in chemical syntheses, in the explosive and dye industry and especially in agriculture as insecticides, herbicides, acaricides and fungicides [1,2]. These compounds are great environmental pollutants since they are usually highly toxic. They accumulate in the body or turn into other compounds with greater toxicity. Numerous fatalities have been described due to their intake [3,4,5,6,7,8]. If their doses accumulate, they remain in the body for approximately 38 days. In soil and water they are microbiologically, but not chemically degradable, with life spans ranging from 8 to 120 days [9].

Recently, dinitrophenol and its derivatives have been used to reduce the symptoms of Alzheimer's, heart and neurodegenerative diseases [10,11,12]. Dinitrophenols act within the respiratory chain as oxidative phosphorylation uncoupling agents. It is assumed that they prevent translocation of protons through the mitochondrial membrane, which leads to the inhibition of oxidative phosphorylation. There are two main theories that try to explain the uncoupling of oxidative phosphorylation from breathing: the chemiosmotic theory [13,14,15,16,17] and the theory according to which dinitrophenols manifest their harmful action due to their capacity to prevent the formation of unstable triplet states [18].

This study assesses the effects of fluorescence quenching in the case of tryptophan with the help of some nitro derivatives [14,19,20,21]. Moreover, the paper aims to study the complexes of dinitrophenols and other related compounds with tryptophan using modern chemical, biochemical and toxicological methods to highlight new aspects of their biological and biomedical activity and obtain new experimental data that can contribute to the elucidation of the mechanisms of action of these highly toxic, antidote-lacking compounds. Thus, the research presented in this paper has the following objectives: the analysis of the biological activity of dinitrophenols compared to that of other inhibitors of oxidative phosphorylation or to that of some stimulants, tests of the fluorescence quenching properties using nitro derivatives, a theoretical study of the physicochemical properties of the studied compounds.

## 2. Results and Discussion

### 2.1. Steady State Fluorescence Studies

Experiments have shown that fluorescence-based analytical methods are very specific and sensitive, therefore these methods can be extremely useful in the analysis and monitoring of certain environmental pollutants. Dinitrophenols and some nitro- and trinitrophenols are able to quench the fluorescence of compounds, therefore in this work we used their interaction with molecules in the excited state to understand the electron and energy transfer from excited molecules to dinitrophenol.

**Figure 1:**
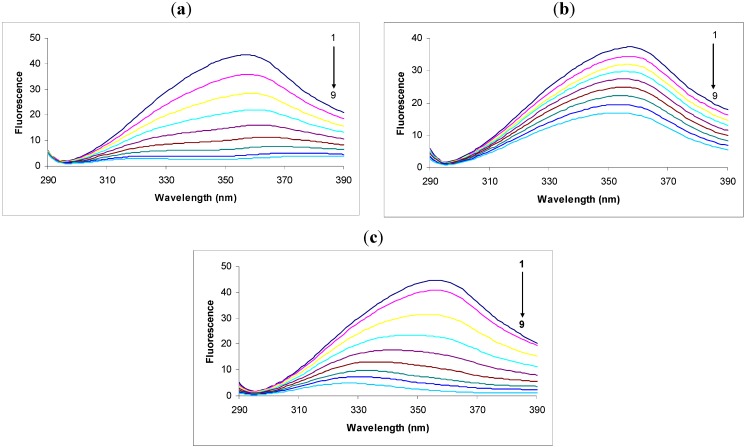
Fluorescence spectra of tryptophan (1.0 μg/mL) in the absence (black, spectrum 1) or presence of: (**a**) 2.4-DNP (6.65 × 10^−6^; 10 × 10^−6^; 13.33 × 10^−6^; 16.66 × 10^−6^; 20.00 × 10^−6^; 23.33 × 10^−6^; 26.66 × 10^−6^; 30.00 × 10^−6^ M, respectively, spectra 2-9); (**b**) 2.6-DNP (6.65 × 10^−6^; 10 × 10^−6^; 13.33 × 10^−6^; 16.66 × 10^−6^; 20.00 × 10^−6^; 23.33 × 10^−6^; 26.66 × 10^−6^ respectively 30.00 × 10^−6^ M, spectra 2-9); (**c**) 2.4-DNOC (6.65 × 10^−6^; 10 × 10^−6^; 13.33 × 10^−6^; 16.66 × 10^−6^; 20.00 × 10^−6^; 23.33 × 10^−6^; 26.66 × 10^−6^ respectively 30.00 × 10^−6^ M, spectra 2-9).

In the fluorescence spectra of tryptophan we observed that the intensity decreased remarkably in response to the addition of 2,4-DNP (Figure 1a). This intensity decrease was accompanied by a large shift and apparent splitting of the maximum emission wavelength from 357 nm to 380 nm and 323 nm in the presence of 30.00 × 10^−6^ M concentrations of 2,4-DNP. The large displacement of the maximum emission wavelength could be due to the hydrophilic or hydrophobic character of the compounds [22,23], thus the theoretical logP was calculated using the specialized Quantitative Structure-Activity Relationship (QSAR) module of the software HyperChem 7.5. Tryptophan has a logP equal to 0.06, 2,4-DNP 1.67 and 2,4-DNP:Tryp complex 0.65. There is a decrease in polarity around tryptophan residues. 

We observed that the intensity of the fluorescence quenching spectra also decreased in response to the addition of 2,6-DNP (Figure 1b). This decrease in intensity was accompanied by a small shift of the maximum emission wavelength from 357 nm to 353 nm in the presence of 30.00 × 10^−6^ M 2,6-DNP. According to the theoretical calculations we performed, 2,6-DNP has the same value of logP as its isomer, but the maximum emission wavelength shift was much smaller, and hence this factor does not have a decisive effect on the maximum emission wavelength shift. Another important factor is probably the heat of formation of the complex with 2,6-DNP, which is higher in this case than in the complex with 2,4-DNP. Therefore, the likelihood of 2,6-DNP:tryptophan complex formation is lower for the same energy consumption.

Studying the spectra of fluorescence quenching in response to the addition of DNOC we observed that the intensity decreased greatly (Figure 1c). Thus, the presence of the compound at a concentration of 30.00 × 10^−6^ M produced a large shift of the maximum emission wavelength from 357 nm to 327 nm. The shifted emission maxima is almost similar with that of 2,4-DNP, both of them having -NO_2_ groups in the same position, and an inductive effect over these overlapped groups was noticed, probably do to an conjugation effect (—E effect). Also, the logP value calculated for DNOC was 2.14 and for the complex it was −1.34, so the complex is soluble in water.

Fluorescence quenching can occur through two different mechanisms, static and dynamic, respectively [24]. We analysed the fluorescence quenching data according to the Stern-Volmer Equation (1):

F_0_/F = 1 + K_SV_[Q] (1)

where F_0_ is the initial intensity of the fluorescent molecule fluorescence signal, F is the fluorescence intensity after adding fluorescence quench molecular solution, K_SV_ is the Stern-Volmer constant and [Q] is the concentration extinguisher fluorescence [21,24]. 

**Figure 2:**
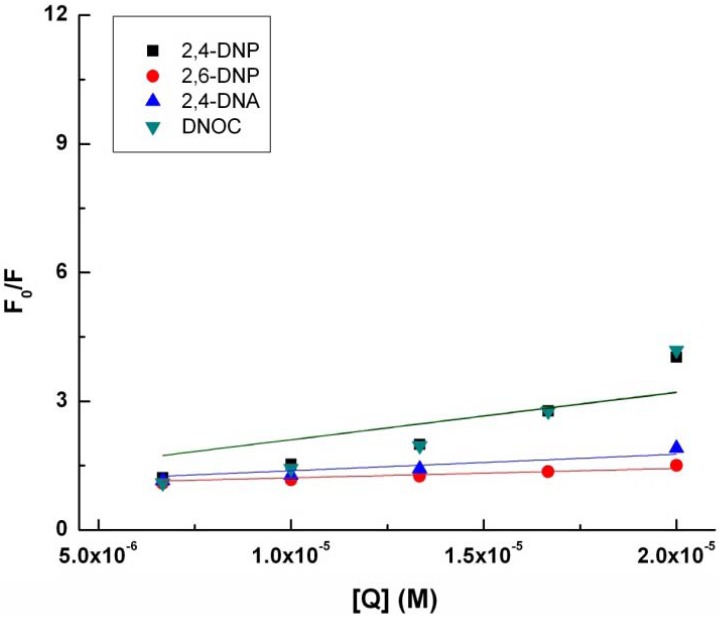
Modified Stern-Volmer plot for the binding of 2.4-DNP, 2.6-DNP, DNA, and DNOC to tryptophan.

With quenching the tryptophan fluorescence in the presence of 2,4-DNP, 2,6-DNP, 2,4-DNA and DNOC, K_SV_, the Stern-Volmer constant increases in the following order: 2,6-DNP, 2,4-DNA, 2,4-DNP and DNOC (Table 1, Figure 2). The difference between the Stern-Volmer constants of 2,4-DNP and DNOC is negligible. In contrast, 2,4-DNA and 2,6-DNP had smaller tryptophan fluorescence quenching constants than 2,4-DNP. From the above results we observe that phenolic hydroxyl blocking or changing the NO_2_ group positions lead to reduced tryptophan fluorescence quenching by dinitrophenol derivatives due to the different resulting electronic effects and conjugation.

**Table 1:** Fluorescence quenching characteristics obtained using linear regression Stern-Volmer Equation (1).

Compound	K_sv_ (M^−1^)	r^2^
2,4-DNP	110116 ± 19365	0.941
2,6-DNP	21760 ± 1731	0.998
2,4-DNA	38270 ± 4723	0.992
DNOC	111048 ± 22833	0.919

Deviations from linearity may exist, they can be positive or negative. If, as in this case, there are positive deviations from the straight line, concave with respect to the Y axis, these may be due to the simultaneous presence of dynamic and static extinction when an excited fluorophore is extinguished due to collision with a fluorescence extinguisher [21,24,25]. Therefore, in these cases, the Stern-Volmer linear regression can be obtained using the Stern-Volmer expresion according to Equation (2):

F_0_/F = 1 + K_SV_ [Q] e^V [Q]^ (2)

where F_0_ is the initial intensity of the fluorescent molecule fluorescence signal, F is the fluorescence intensity after adding fluorescence quench molecular solution, K_SV_ is the Stern-Volmer constant for the quenching of fluorescence by dynamic mechanism, V is a constant representing the static contributions to extinguish fluorescence and [Q] is the concentration of fluorescence extinguisher [26].

By analyzing the data obtained, it was concluded that we must use another Stern-Volmer equation more suitable for our cases [26,27]. Therefore, we studied each representation separately and in accordance with these results Stern-Volmer representations and linear regressions were obtained using the Stern-Volmer expression according to Equation (2). Further, we studied the mechanisms of tryptophan fluorescence quenching by 2,6-DNP, DNOC 2,4-DNA and compared the mechanism of action of 2,4-DNP [27].

**Figure 3:**
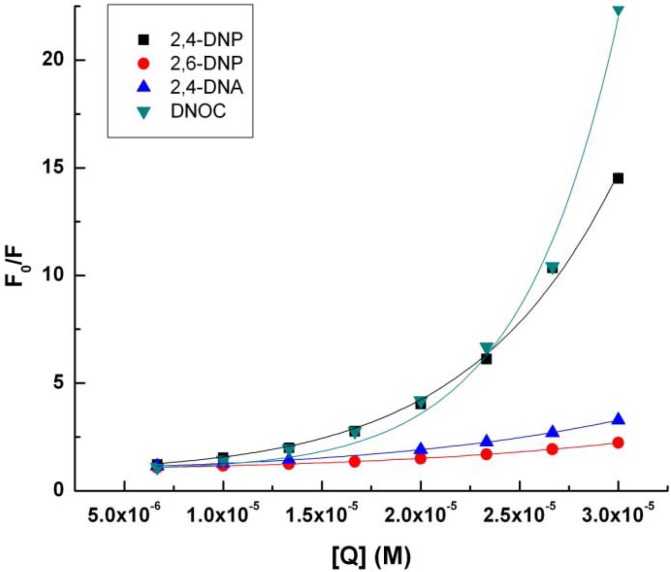
Stern-Volmer representation (2) for tryptophan (4.9 × 10^−3^ M) in presence of 2,4-DNP, 2,6-DNP, 2,4-DNA, DNOC.

Data from the linear regression implementation of Stern-Volmer Equation (2) can be seen in Table 2 and Figure 3.

**Table 2:** Fluorescence extinction characteristics obtained using nonlinear regression Stern-Volmer Equation (2).

Compound	K_SV_ (M^−1^)	V(M^−1^)	r^2^
2,4-DNP	20117.21 ± 3482.26	104272.40 ± 6100.06	0.995
2,6-DNP	9779.84 ± 241.19	47789.49 ± 918.83	0.999
2,4-DNA	16339.86 ± 249.81	51308.83 ± 560.44	0.999
DNOC	4397.68 ± 1574.75	169008.33 ± 12208.50	0.992

K_SV_, the Stern-Volmer constant for the quenching of fluorescence by the dynamic mechanism increases in the following order: DNOC, 2,6-DNP, 2,4-DNA, 2,4-DNP. Surprisingly, DNOC fluorescence quenching by the dynamic mechanism was 80% less compared with 2,4-DNP, although both compounds are very strong. There is a big difference between the value of the constant K_SV_ corresponding to 2,4-DNP compared to the other constants.

If we consider a static quenching mechanism of tryptophan fluorescence, as shown in the table above, V, the Stern-Volmer constant for the quenching of fluorescence by the static mechanism, increases in the following order: 2,6-DNP, 2,4-DNA, 2,4-DNP and DNOC.

If we consider the results in Table 1, Table 2, but also properties of a QSAR study of the 2,4-dinitro derivatives and their complexes with tryptophan, then a decreased tryptophan fluorescence quenching capacity through dynamic mechanism occurs with increasing volume (see the section on theoretical calculations). 2,4-DNP has a greater capacity for tryptophan fluorescence quenching that 2,6-DNP, probably due to the conjugation that arises from the position of the nitro group *para* to the phenolic hydroxyl group.

### 2.2. Spectroscopic Studies on Some Dinitrophenol and Dinitrophenyl Ethers

**Figure 4:**
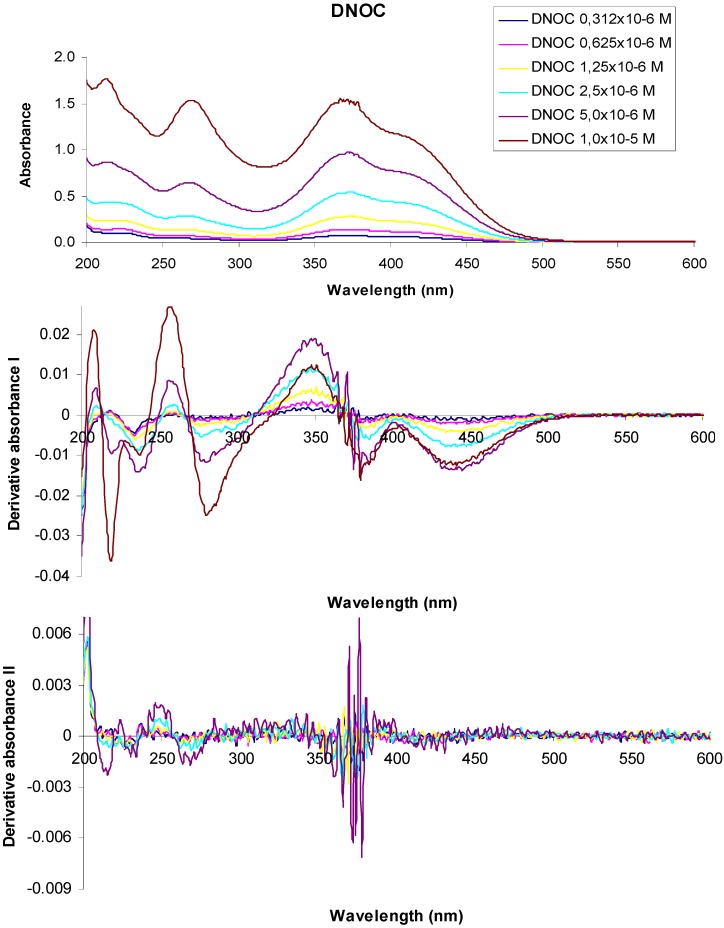
UV-VIS absorption spectra of 3,5-dinitro-*o*-cresol and their derivatives at various concentrations.

To elucidate the action of mono- and dinitro derivatives on the oxidative phosphorylation mechanism we studied the fluorescence quenching of tryptophan or other compounds in the presence these uncoupling agents. Figure 4 shows the absorption spectra and derivative curves of some representative dinitrophenol ethers and dinitrophenols.

In the UV-VIS absorption spectrum of a 1 × 10^−5^ M aqueous solution of 3,5-dinitro-*o*-cresol (DNOC), we observed three absorption maxima at 213 nm (A = 1.776), 268 nm (A = 1.537), 367 nm (A = 1.550) and two shoulders at 229 nm (A = 1.396) and 415 nm (A = 1.107). The UV-VIS absorption spectrum of a 5 × 10^−6^ M DNOC solution presented three absorption maxima at 213 nm (A = 0.868), 268 nm (A = 0.644), 372 nm (A = 0.975) and two shoulders at 230 nm (A = 0.752) and 416 nm (A = 0.720). Using 2.5 × 10^−6^ M DNOC concentration we observed three absorption maxima at 215 nm (A = 0.435), 266 nm (A = 0.285), 372 nm (A = 0.541) and a shoulder at 417 nm (A = 0.419). In the UV-VIS absorption spectrum of DNOC solutions of 1.25 × 10^−6^ M, 6.25 × 10^−7^ M and 3.12 × 10^−7^ M concentration, we observed three absorption maxima at 220 nm (A = 0.237), 264 nm (A = 0.139), 374 nm (A = 0.282) for the first solution, three absorption maxima at 220 nm (A = 0.144), 264 nm (A = 0.073), 377 nm (A = 0.141) in the case of the second solution and three absorption maxima from 222 nm (A = 0.105), 264 nm (A = 0.045), 376 nm (A = 0.080) in the case of the third solution, respectively.

The first order derivative of the 1 × 10^−5^ M DNOC solution spectra intersects the axis at wavelengths equal to 213 nm, 270 nm, 366 nm, at a concentration of 5 × 10^−6^ M, at 213 nm, 267 nm, 369 nm, at a concentration of 2.5 × 10^−6^ M, at 216 nm, 265 nm, 370 nm, at a concentration of 1.25 × 10^−6^ M at 217 nm, 264 nm, 369 nm, at a concentration of 6.25 × 10^−^^7^ M, at 212 nm, 263 nm, 368 nm and at a concentration of 3.12 × 10^−^^7^ M, at 215 nm, 264 nm, 368 nm respectively.

In the second order derivative of the spectra of a 1 × 10^−5^ M DNOC solution, the minimum is observed more at 215 nm, 234 nm, 267 nm, 365 nm, 370 nm, 379 nm, of which the most important are at 215 nm, 267 nm, 370 nm. If the concentration is 5 × 10^−6^ M, the minimum is observed more at 215 nm, 230 nm, 233 nm, 263 nm, 268 nm, 361 nm, 366 nm, 372 nm, 374 nm, 376 nm, 378 nm, among which the most important is at 215 nm, 267 nm, 378 nm; If the concentration is 2.5 × 10^−6^ M, at 216 nm, 233 nm, 263 nm, 268 nm, 360 nm, 365 nm, 369 nm, 374 nm, 376 nm, 378 nm, of the most important is at 215 nm, 267 nm, 378 nm. For the the concentration of 1.25 × 10^−6^ M, at 377 nm and when the concentration is 6.25 × 10^−^^7^ M, the minimum is observed at 376 nm.

Radiant energy absorbed by one mole of substance, E, was calculated using the following equation:

E = 28635/λ_max_ (3)

where E is the energy, λ_max_ is the wavelength at which maximum power is calculated [28]. Studying Table 3, it is noted that 2,4-DNP has the largest absorbed radiant energy.

**Table 3:** Average absorbance and radiant energy absorbed by one mole of substance calculated for 1 M solutions of nitro compound.

Name of substance	λ_max_	Average absorbance at concentration of M × 10^−5^	E kcal/mol
2,4-DNP	212	2.55	135.02
	259	2.10	110.56
	359	2.88	79.76
2,6-DNP	222	2.58	128.99
	255	0.89	112.29
	432	0.87	66.39
2,4-DNA	213	1.74	134.44
	260	1.21	110.13
	299	1.26	95.77
DNOC	213 (2.5−10.0 ×10^−6^ M)	1.74	134.44
	268 (2.5−10.0 ×10^−6^ M)	1.31	108.47
	372 (2.5−10.0 ×10^−6^ M)	1.88	76.98

### 2.3. Theoretical Studies

We looked for possible correlations between these parameters and optical properties of the biological compounds investigated. We studied the molecular parameters of dinitrophenol and dinitrophenyl ethers parameters compared with other compounds or their analogues showing similar properties. 

**Figure 5:**
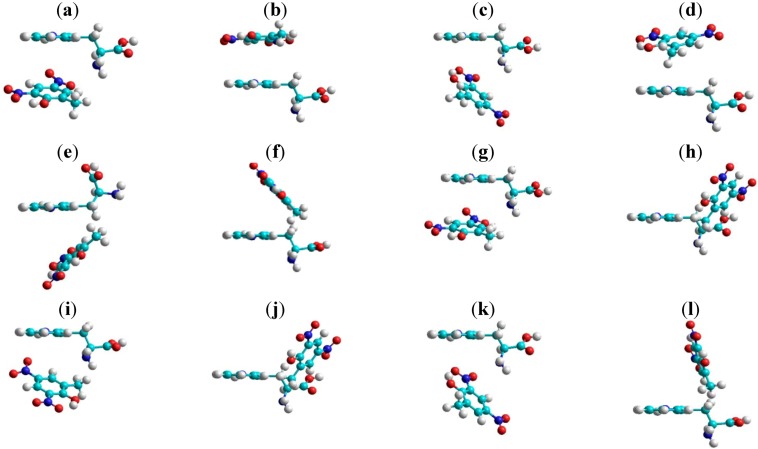
DNOC:Tryptophan structures optimized by the PM3 method.

According to PM3 and fluorescence quenching methods the volume, refractivity and polarizability can be correlated with the quenching constants. The tryptophan conformers were analyzed in the first step, then the most stable variants were chosen. Only the 3,5-dinitro-*o*-cresol:tryptophan complex is discussed as an example (Figure 5). Some molecular properties of dinitro-derivative:tryptophan complexes calculated by the MM+ and PM3 methods are presented for comparison in Table 4. Variation of energy complexes calculated by the PM3 methods are presented for in Table 5. For a deeper study of the properties of molecular complexes with dinitro-derivatives and tryptophan we studied the most stable structures.

If we take into account the electronic chemical potential of 2,4-DNP compared with that of 2,4-DNA, respectively, at the DNOC site it is noted that 2,4-DNP has lower electronic chemical potential (Table 6).

**Table 4:** QSAR properties of complexes calculated by MM+ and PM3 methods.

**Complex Methods**	**2.4-DNP:Trip**		**DNOC:Trip**		**DNAN:Trip**	
	MM+	PM3	MM+	PM3	MM+	PM3
Volume Å^3^	919.12	1037.42	965.51	1038.70	960.24	1084.00
Refractivity Å^3^	95.09	95.09	99.57	99.57	99.03	99.99
Polarizability Å^3^	36.82	36.82	38.66	38.66	38.66	38.66

**Table 5:** Variation of energy complexes PM3.

Complex	ΔTotal Energy	ΔBinding Energy	ΔHeat of Formation
	kcal/mol	kcal/mol	kcal/mol
2,4-DNP:Tryp	−24.79	−30.78	−24.79
2,4-DNOC:Tryp	−28.85	−27.85	−28.85
2,4-DNA:Tryp	−38.05	−38.05	−38.06

**Table 6:** Energy data calculated theoretically PM3.

Name of substance	E_HOMO_	E_LUMO_	ΔE	*μ*=-χ_M_	*η*	Δ *N*
	_eV_	_eV_	_eV_	_eV_	_eV_	
2,4-DNP	−10.79	−1.88	−8.91	−6.33	−4.46	0.22
2,6-DNP	−10.66	−1.99	−8.67	−6.33	−4.33	0.22
2,4-DNA	−10.81	−1.69	−9.12	−6.25	−4.56	0.21
DNOC	−10.67	−1.75	−8.92	−6.21	−4.46	0.21

If the most stable complexes were obtained the results are as summarized in the following table:

Comparing the data summarized in Table 6, Table 7 we observe that for the studied compounds the electronegativity values differ greatly from those of the corresponding complexes. The electronic chemical potential value of the 2,4-DNP-tryptophan complex *versus* the electronic chemical potential values corresponding to the other complexes is higher for the derivatives, therefore, the electronegativity is the smallest. The electronic chemical potential could make important contributions to the elucidation of the mechanism of toxicity of 2,4-DNP community. We sought possible correlations between these parameters and the optical or biological properties of the investigated compounds [29,30]. Analysis was carried out about the physicochemical properties of mono-complexes and dinitro-derivatives with tryptophan using Pearson’s reactions [31]. 

**Table 7:** Theoretical energy data calculated using the HyperChem program PM3 method.

Name of substance	E_HOMO_	E_LUMO_	ΔE	*μ*=-χ_M_	*η*	Δ *N*
	_eV_	_eV_	_eV_	_eV_	_eV_	
2,4-DNP:Tryp	−8.55	−1.40	−7.15	−4.98	−3.57	0.22
2,6-DNP:Tryp	−8.67	−1.94	−6.74	−5.31	−3.37	0.22
2,4-DNA:Tryp	−8.81	−1.56	−7.24	−5.18	−3.62	0.21
DNOC:Tryp	−8.58	−1.76	−6.82	−5.17	−3.41	0.21

### 2.4. Toxicological Studies

**Figure 6:**
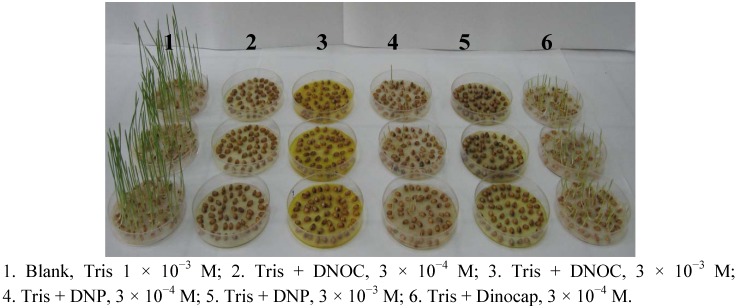
Effect of 4,6-dinitro-*o*-cresol (DNOC), 2,4-dinitrophenol (2,4-DNP) and dinocap on wheat seed germination.

We studied the biological activity of some dinitrophenols on the germination of wheat seeds. The results were statistically interpreted [32]. We thus studied the biological effects of DNOC, 2,4-DNP and dinocap on the germination of wheat seeds of the variety Gasparom compared to a control sample of 1 × 10^−3^ M Tris solution at pH 7 and 22 °C temperature. Treatment solutions had the same concentration as blank Tris (Figure 6). The toxicity of DNOC and 2,4-DNP at a concentration of 3 × 10^−3^ M is 100%. At a concentration of 3 × 10^−4^ M they inhibited germination—dinocap by 19%, 2,4-DNP by 87% and DNOC by 99%. Comparing the results of these treatments, it appears that at the same concentration DNOC is the most toxic and dinocap the least toxic. In the case of DNOC no plantlets were obtained (Figure 6).

If we consider the total height of the plantlets in a lot, the plantlets’ mass in a lot and the average mass of plantlets, 2,4-DNP reduced the total height of the plantlets by 97.3%, the plantlets’ mass in a lot by 96.4% and the average mass of plantlets by 61.5% and dinocap reduced the total height of the plantlets in a lot by 76.9%, the plantlets’ mass in a lot by 36.3% and the average mass of plantlets by 54.7%. According to the Tukey test differences were significant compared to the blank, with one exception, germination of sample 6. The toxicity of 3 × 10^−4^ M concentrations increases in the following order: mixture of Tris with dinocap is less toxic than the mixture of Tris with DNP, which is less toxic than the mixture of Tris with DNOC. 

## 3. Experimental

### 3.1. Materials

The reagents used were of analytical purity (obtained from Merck, Darmstadt, Germany; Sigma-Aldrich, Taufkirchen, Germany; Roth, Karlsrhue, Germany or Chimopar, Bucharest, Romania). 

### 3.2. Biological Material

Gasparom variety wheat seeds were produced by the Agricultural Research and Development Station Suceava.

### 3.3. Fluorescence Measurements

To study the fluorescence quenching fluorescence spectra were recorded on a Kontron SFM-25 (Kontron Instruments SPA, Milan, Italy) instrument equipped with quartz cuvettes with a path length of 1 cm with total volume of 3 mL and a thermostat or a LIBBRA S35 PC (Biochrom, Cambridge, UK) spectrophotometer equipped with 1 cm quartz cuvettes (Hellma, Müllheim, Germany) with total volume of 3 mL and a thermostat. Samples were excited at λ_ex_ = 380 nm. Initially we prepared 20 mM Tris buffer solution of pH 7 by dissolving 120 mg of Tris in 30–40 mL water. The pH was adjusted to pH 7 with hydrochloric acid. We obtained the tryptophan stock solution by dissolving 20 mg of tryptophan in 50 mL water. 200 mL of this solution were pipetted into 50 mL Tris buffer of a pH 7 and, thus, we obtained a solution of 4.9 × 10^−3^ M tryptophan. The fluorescence quenching measurements were made with stock solutions of concentration 1 × 10^−3^ M nitro compound. Volumes of 20, 30, 40, 50, 60, 70, 80 and 90 μL obtaining final solution concentrations of 6.65 × 10^−6^, 1 × 10^−5^, 1.333 × 10^−5^, 1.666 × 10^−5^, 2.000 × 10^−5^, 2.333 × 10^−5^, 2.666 × 10^−5^ and 3.000 × 10^−5^ M, respectively.

### 3.4. Calculation Details

We performed MM+ and PM3 calculations using HYPERCHEM release 7.5 for Windows 2007. We created the molecular models using a PC Intel (R) Core (TM) 2 Duo CPU T5450, 997 MHz, 1024 MB RAM computer. In molecular mechanics we used the MM+ force field. For the optimization we used the Polack Ribiere Algorithm with a RMS gradient of 0.001 kcal/mole. The tryptophan conformers were analyzed in the first step, then the most stable variants were chosen. Using HyperChem 7.5 program 77 conformers were obtained.

### 3.5. Toxicological Studies

The germination parameters were measured according to ISTA recommendations (Seed Science and Technology, 1993) [14,19]. We worked with lots of 50 seeds which were laid to germinate on filter paper, in Petri dishes, in three replicates. We did the first count after three days (energy of germination, EG), the second after 7 days (germination rate, GR). The germinated, abnormal and dead seeds as well as the resulting plantlets were counted. The treatments lasted for one hour, followed by the uniform distribution of the seeds in the Petri dishes, on double filter paper, together with the treatment solutions. The seeds with a visible root were considered germinated. The seeds were watered daily with 5 mL of redistilled water. The plantlets were cut at the level of the seeds 7 days later, measured and weighed (height, H, in cm and mass, m, in grams). The results were processed using the Tukey test [32]. The mean square deviation s_x_ of the samples was also calculated, as well as t factor, with a view to compare the results obtained under the action of different treatments.

## 4. Conclusions

The research conducted in this paper allows us to draw the following general conclusions: the spectroscopic studies of some nitro-derivatives led to the conclusion that the highest value of the radiant energy absorbed by one mole of substance, E = 26835/λ_max_ (kcal/mol) was obtained in the case of 2,4-DNP.

The following considerations can be made about the ability of fluorescence quenching of dinitrophenols and their derivatives: the high values of the quenching constants provide proof of the high efficiency of dinitrophenols and their derivatives as fluorescence quenchers; out of the studied compounds, 2,4-DNP and DNOC showed the highest values of tryptophan fluorescence quenching constants, these being also the most toxic compounds. Decreased tryptophan fluorescence intensity in the presence of mono- or dinitro-derivatives is generally accompanied by a shift in the maximum emission wavelength. 2,4-DNP and DNOC, which are the most powerful quenchers of tryptophan fluorescence, produced the biggest variations in maximum emission wavelengths. The lack of -OH phenolic group or its replacement decreased the tryptophan fluorescence quenching ability by dinitro-derivatives. The change of NO_2_ group from position 4 also led to the decrease in the values of Stern-Volmer constants. Nitro-derivatives acted through the dynamic mechanism as well as through the static mechanism of tryptophan fluorescence quenching. 

The theoretical studies of some dinitro-derivatives led to the following conclusions: in the theoretical study of the interaction of dinitro-derivatives with tryptophan by the MM+, PM3 and AM1 methods, we obtained the values of QSAR properties (the accessible surface of the solvent, the molecular volume, logP, refractive and polarization ability) for the complexes. The interaction energy was calculated using the MM+ method and ΔH_association_ and the dipole moment for the studied complexes were calculated using the PM3 method. The value of the electronic chemical potential of the most stable complex of 2,4-DNP with tryptophan is higher than the values of the electronic chemical potentials of complexes corresponding to derivatives.

The experiments with germinating wheat seeds demonstrated their sensitivity to dinitrophenols and dinitrophenyl ethers, in the concentration range of 10^−4^ M to 10^−2^ M. Dinitrophenols are highly toxic compounds that do not allow the germination of wheat seeds starting with the concentration of 3 × 10^−3^ M. The experimental results obtained were accurate and statistically secured.

The mechanism of action of the investigated compounds appears to be consistent with the well-known bio-structural theory developed by Macovschi, while the data obtained indicate the need for reassessing Mitchell’s chemiosmotic hypothesis on ATP synthesis during the breathing process as a result of proton translocation through mitochondrial membranes.

The data obtained open the way for further research to evaluate the relationship between the chemical structure of dinitrophenols as well as of other derivatives, and their properties as uncoupling agents. Further investigations are needed to provide further explanations and details of the oxidative phosphorylation mechanisms.
